# dsRNA-Mediated Pest Management of *Tuta absoluta* Is Compatible with Its Biological Control Agent *Nesidiocoris tenuis*

**DOI:** 10.3390/insects12040274

**Published:** 2021-03-24

**Authors:** Nomi Sarmah, Athanasios Kaldis, Clauvis Nji Tizi Taning, Dionysios Perdikis, Guy Smagghe, Andreas Voloudakis

**Affiliations:** 1 Laboratory of Agricultural Zoology and Entomology, Agricultural University of Athens, 11855 Athens, Greece; sarmahnomi3@gmail.com (N.S.); dperdikis@aua.gr (D.P.); 2 Laboratory of Plant Breeding and Biometry, Agricultural University of Athens, 11855 Athens, Greece; akaldis2003@hotmail.com; 3 Laboratory of Agrozoology, Department of Plants and Crops, Faculty of Bioscience Engineering, Ghent University, 9000 Ghent, Belgium

**Keywords:** RNA interference (RNAi), biosafety, integrated pest management (IPM), biological control, oral droplet feeding

## Abstract

**Simple Summary:**

The zoophytophagous mirid bug *Nesidiocoris tenuis* is an efficient predator of the tomato leafminer, *Tuta absoluta.* RNA interference (RNAi) targeting the *alphaCOP* (*αCOP*) (Coatomer subunit alpha protein) gene of *N. tenuis* (*Nt-αCOP*) was proven to be functional in *N. tenuis*, causing downregulation of gene expression, mortality and sub-lethal effects. In contrast, when *N. tenuis* were fed with dsRNA (ds*Ta*-*αCOP*) targeting the ortholog *αCOP* gene of *T. absoluta*, no lethal nor sub-lethal effects were observed. These results indicate the compatibility of this biocontrol agent along with RNAi-mediated management in order to suppress *T. absoluta* efficiently in tomato crop.

**Abstract:**

RNAi-mediated insect pest management has recently shown promising results against the most serious pest of tomato, the tomato leafminer, *Tuta absoluta*. This study aimed to investigate whether dsRNA (ds*Ta*-*αCOP*) designed to target the *T. absoluta-αCOP* gene could cause adverse effects to its biocontrol agent, the mirid predator, *Nesidiocoris tenuis.* Oral exposure of *N. tenuis* to dsRNA (ds*Nt-αCOP*) designed to target *N. tenuis*-*αCOP* resulted in a 61%, 67% and 55% reduction in its transcript level in comparison to the sucrose, ds*GFP* and ds*Ta-αCOP* treatments, respectively. In addition, significantly higher mortality of 57% was recorded in ds*Nt-αCOP-*treated *N. tenuis* when compared to the sucrose (7%), ds*GFP* (10%) and ds*Ta-αCOP* (10%) treatments. Moreover, the predation rate of ~33–39 *Ephestia kuehniella* eggs per *N. tenuis* adult dramatically reduced to almost half in the surviving ds*Nt-αCOP-*treated *N. tenuis*. This worst-case exposure scenario confirmed for the first time that the RNAi machinery is functional in this species and that the risk of exposure through the oral route is possible. In contrast, ds*Ta*-*αCOP* did not cause any sub-lethal effects to *N. tenuis* upon oral exposure. Oral exposure of *T. absoluta* to ds*Ta*-*αCOP* resulted in 50% mortality. In the context of a biosafety risk assessment of RNAi-mediated insect management, investigating the effects on non-target organisms is essential in order to include this method as part of an integrated pest management strategy. Based on our laboratory assays, RNAi-mediated control is compatible with the biological control of *T. absoluta* by its natural enemy *N. tenuis*, adding the RNAi approach in the armoire of integrated pest management of *T. absoluta*.

## 1. Introduction

The tomato (*Lycopersicon esculentum*) is one of the most important food and cash crops worldwide. Τhe tomato leafminer, *Tuta absoluta* (Meyrick) (Lepidoptera, Gelechiidae), is one of the most devastating tomato pests, which may cause up to 100% yield loss [1,2]. The voracious generalist predator *Nesidiocoris tenuis* (Reuter) (Hemiptera, Miridae) is a major biological control agent reported in tomato plants and is widely used against *T. absoluta* [3,4]. *N. tenuis* may reduce up to 56% the leaflet infestation of *T. absoluta* by actively searching and feeding on *T. absoluta* eggs and larvae in glasshouse condition [5,6,7]. However, apart from its beneficial role, *N. tenuis* may cause damage to the plant due to its phytophagy [8,9]. For this reason, the release of a lower number of *N. tenuis* is highly recommended [6]. Another drawback of using *N. tenuis* is the need of extended establishment period on the crop [9]. Although, *N. tenuis* is a reliable contributor to *T. absoluta* management, these limitations may hamper its efficacy and further its adoption by the farmers. Consequently, aiming to sustain the benefits of the biological control and increase the tomato crop production, the possibility to combine biological control with another compatible pest control method must be explored.

In search for an environmentally friendly approach to manage *T. absoluta*, the RNA interference (RNAi)-based control strategy was recently employed with encouraging results [10,11]. RNAi refers to the sequence-specific process found in insects and other eukaryotic organisms, which is initiated by the presence of double-stranded RNA (dsRNA) and leads to the regulation of target messenger RNAs (mRNAs) via post-transcriptional knockdown pathways [12]. DsRNA-specific endonucleases, such as Dicer-2, enable in the production of small interfering RNAs (siRNAs) from the dsRNA. In turn, siRNAs are loaded to the RNA-induced silencing complexes (RISCs), having as a central component the Argonaute proteins, which possess endonucleolytic capacity. Argonautes use the siRNAs as guides for the recognition of mRNAs with matching sequence identity, finally causing the degradation or the translational inhibition of these mRNAs. The RNAi pathway in insects is triggered as a response to exogenous dsRNA, and is mediated through Dicer-2, Argonaute-2 and the insect-specific dsRNA-binding protein R2D2. 

Phylogenetic analysis in insects revealed that the aforementioned core components of RNAi might have been duplicated or lost during the evolutionary history. This could explain the observed variability in the efficacy of RNAi across different insect lineages [13]. The pioneering work in the field of RNAi was the report of a substantial decline of an endogenous mRNA transcript through the injection of homologous dsRNA in *Caenorhabditis elegans* (Maupas) (Rhabditida, Rhabditidae) [14]. The first RNAi-mediated effects in insects were recorded in the fruit fly *Drosophila melanogaster* Meigen (Diptera, Drosophilidae) [15]. Since then, application of RNAi mechanism has been extensively studied in insects [16,17].

In insect pest control, RNAi-mediated gene knockdown effect has been demonstrated in Diptera, Coleoptera, Hymenoptera, Orthoptera, Blattodea, Lepidoptera, and Hemiptera [18,19,20,21]. Among Lepidoptera, satisfactory RNAi results have been reported in *Spodoptera exigua* (H.) (Noctuidae) [22] *Helicoverpa armigera* (H.) (Noctuidae) [23] *Plutella xylostella* (L.) (Plutellidae) [24], and *Mythimna separata* (W.) (Noctuidae) [25]. Regarding *T. absoluta*, functional RNAi was achieved in several genes such as Juvenile hormone inducible protein (*JHP*), Juvenile hormone epoxide hydrolase protein (*JHEH*), Ecdysteroid 25-hydroxylase (*PHM*), Chitin synthase A (*CHI*), Carboxylesterase (*COE*) [11], Arginine kinase (*AK*) [10,11], and Vacuolar ATPase-A, which exhibited significant larval mortality in this pest [11,26]. 

Besides its efficacy, integrating RNAi in Integrated Pest Management (IPM) strategies requires the assessment of RNAi’s effects on the natural enemies of the target pests. Since RNAi is a sequence-based mechanism, any dsRNA with sufficient homology to an arthropod mRNA may trigger RNAi in a non-targeted manner. Therefore, dsRNA-mediated approaches under development should be accompanied with ecotoxicological studies to identify any effects in non-target organisms [27,28,29,30]. Castellanos et al. [31] reported that the combined use of RNAi-mediated gene silencing against the Neotropical brown stink bug *Euschistus heros* (F.) (Hemiptera, Pentatomidae), together with eggs of the parasitoid *Telenomus podisi* Ashmead (Hymenoptera, Scelionidae), proved to be a feasible approach. In RNAi-mediated pest control, off-target effects need to be analysed before its implementation [32,33]. In a study by Pan et al. [28], the effects of dietary RNAi toxicity assay of *Diabrotica virgifera virgifera* LeConte (Coleoptera, Chrysomelidae) active dsRNA for the gene *V-ATPase* proved to be detrimental to four non-target ladybird species (Coleoptera, Coccinellidae). These studies denote the critical need to evaluate the lethal or sub-lethal effects of RNAi approach on natural enemies as an essential aspect for the decision of incorporating RNAi in IPM programs. 

The main objective of the present study was to investigate whether the RNAi-based control of *T. absoluta* may cause lethal or sub-lethal effects on its major natural enemy, namely the predator *N. tenuis*. We present, for the first time, evidence that the performance of the predator *N. tenuis* is not negatively affected by the dsRNA targeting the ortholog *αCOP* gene of *T. absoluta*. This raises the possibility of combining RNAi-mediated management with the biological control of *T. absoluta* for the effective protection of tomato crops from this notorious pest. 

## 2. Materials and Methods

### 2.1. Insect Rearing and Maintenance

Tomato plants (cv. Elpida) were developed from seeds without applying any pesticide. They were inspected daily, and any pest found was removed. *Nesidiocoris tenuis* (Nesibug™, Koppert, Berkel en Rodenrijs, the Netherlands) were reared on tomato plants and were provided with a mixture of *Ephestia kuehniella* Zeller (Lepidoptera, Pyralidae) eggs and dried cysts of *Artemia* sp. (Crustaceae) (Entofood™, Koppert) *ad libitum,* twice per week. *T. absoluta* initially collected from Marathon, Greece (Latitude: 38°09’11.41” N, Longitude: 23°57’46.01” E) were reared on tomato plants. Adult moths were fed with sugar solution (10% sucrose); a piece of cotton was half-dipped in the sugar solution in each of four plastic cups (30 mL), placed inside a rearing cage. Adult moths could feed from the piece of cotton half-hanging outside each cup. Each rearing was maintained separately in wooden entomological cages (80 × 80 × 70 cm) in a glasshouse at temperature of 25 ± 2.5 °C and natural lighting at the Agricultural University of Athens, Greece.

### 2.2. Target Gene Selection and dsRNA Synthesis

As a target gene for RNA silencing in *T. absoluta,* the *alphaCOP* (*αCOP*) (Coatomer subunit alpha protein) was selected. *αCOP* is an essential eukaryotic gene responsible for the mediation of biosynthetic protein transport from the endoplasmic reticulum to the Golgi network. To investigate the risk of exposure of *N. tenuis* to dsRNA targeting *T. absoluta*, namely *Ta-aCOP*, a worst-case scenario was considered where *N. tenuis* was directly exposed to dsRNA designed to specifically target *Nt*-*aCOP*. To identify the nucleotide sequence of *αCOP* in *N. tenuis,* the *αCOP* sequence from *Halyomorpha halys* (Stål) (Hemiptera, Pentatomidae) (Accession no. XM_014431769.2) was used as a “query” against the whole-genome shotgun contig database of NCBI via BLASTN analysis. A corresponding genome shotgun sequence from *N. tenuis* (Accession no. CADCXU010020467.1) was used to design primers for the synthesis of dsRNA molecules of *Nt-aCOP* (Table 1). 

To produce ds*Nt-αCOP*, total RNA was extracted from the fifth instar nymphs of *N. tenuis* using a TRIzol-based method essentially as described by Yoo et al. [34]. A number of pooled nymphs were frozen in liquid nitrogen and then added in an Eppendorf tube containing 500 μL TRIzol reagent (Ambion, Austin, TX, USA). Quickly, we ground the insect tissue inside TRIzol using a homogenizer with suitable pestles for 10 min. Then we placed the tube at 4 °C for 1 h. We added 100 μL of chloroform, mixed well and centrifuged at 15,000× g for 15 min. RNA was then precipitated using isopropanol and sodium acetate. After drying, the RNA pellet was diluted in RNase-free H_2_O. The optical density (OD) at 260 nm was measured spectrometrically by a Fisher Scientific Multiskan FC Reader. Residual genomic DNA from the extracted RNA was removed by Turbo DNA free kit (Ambion, Austin, TX, USA), after which cDNA was prepared from the RNA, using oligo-dT and Superscript IV (Thermo Fisher Scientific, Carlsbad, CA, USA). A selected fragment of *Nt-αCOP* was amplified by PCR (95 °C—2 min, 35 cycles of 95 °C—30 s, 60 °C—30 s, 72 °C—45 s, followed by 72 °C—5 min) with *Taq* DNA polymerase (Invitrogen,**** Waltham, USA), using 500 ng of cDNA as a template and *Nt-αCOP* specific primers flanked at their 5′ ends by a T7 promoter sequence (5′-TAATACGACTCACTATAGGG-3′) (Table 1). The identity of the *Nt-αCOP* fragment was verified by sequencing. In vitro synthesis of dsRNA targeting the *αCOP* gene of *N. tenuis* (ds*Nt*-*αCOP*) was then performed using the MEGAscript RNAi kit (Thermo Fisher Scientific) essentially as described by [35]. The length of ds*Nt-αCOP* was 391 nt (Figure S1). As a negative control, dsRNA targeting the *GFP* (Green Fluorescent Protein of jellyfish *Aequorea victoria* gene) (ds*GFP*), a gene that is absent in insects, was also synthesized using a plasmid containing a GFP insert (NC_011521.1) and GFP-specific primers (TACGGCGTGCAGTGCT, TGATCGCGCTTCTCG). The length of ds*GFP* was 455 nt (Figure S1).

To identify the nucleotide sequence of *Ta-αCOP*, the *αCOP* sequence from *Papilio xuthus* (L.) (Lepidoptera, Papilionidae) (Accession no. XM_013305848.1) was used for BLASTN analysis. A corresponding genome shotgun sequence from *T. absoluta* (Accession no. SNMR01042501) was used to design primers for dsRNA production. DsRNA targeting *Ta-αCOP* (ds*Ta-αCOP*) was synthesized as described above for *Nt-αCOP*. The length of ds*Ta-αCOP* was 505 nt (Figure S1). The concentration of all dsRNAs produced was brought to 1 μg/μL using a Fisher Scientific Multiskan FC Reader (Thermo Fisher Scientific).

### 2.3. Oral Delivery of dsRNA to N. tenuis and T. absoluta

**Figure 1:**
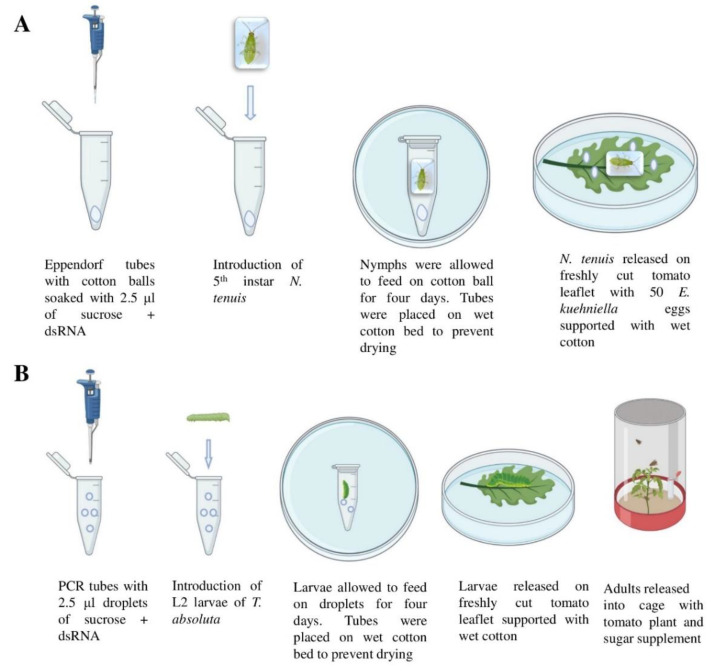
Experimental setup for oral delivery of dsRNA via sucrose. (**A**) For *N. tenuis* setup, cotton balls were soaked with a solution composed of 0.5 M sucrose + 0.5 μg/μL dsRNA in an Eppendorf tube. A single fifth instar nymph was introduced in each Eppendorf tube for 4 d. Three independent experiments employing 14 Eppendorf tubes were performed for each treatment. Eppendorf tubes containing sucrose only (without dsRNA) were used as the control treatment. (**B**) In *T. absoluta*, ten 0.25 μL droplets (total amount 2.5 μL) of a solution composed of 0.5 M sucrose + 0.5 μg/μL dsRNA were placed on the inner wall of the Polymerase Chain Reaction (PCR) tube. A single L2 larva was introduced in each PCR tube, remaining there for 4 d. Three independent experiments employing 14 PCR tubes were performed for each treatment. PCR tubes containing sucrose only (without dsRNA) were also included as control treatment. Image was created using biorender.com (accessed on 4 February 2021).

The oral feeding method [28,36,37] was used to deliver ds*Nt-αCOP*, ds*GFP,* ds*Ta-αCOP* and sucrose solution to *N. tenuis*. In brief, a 1.2 mg cotton ball was inserted into a sterile 1.5 mL Eppendorf tube and was soaked with a sucrose droplet containing either 2.5 μL of sucrose solution (0.5 M) or 2.5 µL of sucrose solution (0.5 M) mixed with specific dsRNA molecules (0.5 µg/µL) (Figure 1A). Second or third instar nymphs were transferred from the rearing cage to caged tomato plants with eggs and cysts offered *ad libitum*, and kept at 25 ± 1 °C, 65 ± 5% RH and 16 h light. Fifth (5th) instar nymphs of *N. tenuis* of 24–48 h in that instar were collected from the caged plants and placed individually in Petri dishes for 2 hrs before their use in the experiments having access only to a tomato leaflet to reduce variation in their hunger level. Then, each nymph was introduced individually into an Eppendorf tube. The tube opening was covered with parafilm and pin holes were made on it to allow adequate ventilation. A preliminary experiment with 30 *N. tenuis* individuals feeding on sucrose only for 4 d was performed. Monitoring for 14 days showed that all individuals survived and moulted to the next instar, indicating that this set up was suitable for the normal development of the predator. The next step was to expose *N. tenuis* to four treatments: (a) sucrose only, (b) sucrose+ds*GFP*, (c) sucrose+ds*Ta-αCOP*, (d) sucrose+ds*Nt-αCOP*. *N. tenuis* nymphs, inside the Eppendorf tubes, could feed on the cotton balls for 4 d (25 ± 1°C, 65 ± 5% RH, and 16 L:8 D photoperiod). The tubes were placed on a wet cotton bed in a 12-cm-diameter Petri dish to prevent the soaked cotton from drying out during the exposure period. The Petri dishes had two mesh-covered holes (3 cm diameter each) on their lids to reduce the accumulation of humidity.

Upon completion of the four-day-feeding period all the nymphs (*n* = 10) from each treatment had moulted to adults. After removal from the tubes, the adults were immediately introduced into individual Petri dishes with a tomato leaflet supported by wet cotton. On the leaflet, 50 eggs of *E. kuehniella* (Epheggs™, Bioinsecta, Thessaloniki, Greece) were offered as prey (Figure 1A) under the above conditions. The number of eggs consumed by *N. tenuis* were counted under a stereoscopic microscope after 24 h, i.e., at 1 dpt (day post-treatment). Then, adults were kept individually in a Petri dish where they were provided daily with a fresh tomato leaflet and Entofood *ad libitum*. Their predation rate on *E. kuehniella* eggs was further assessed at 4 dpt. The mortality of *N. tenuis* individuals was monitored every day up to 10 dpt. The experiment was repeated thrice. 10 nymphs/treatment were used in each experiment. 

In *T. absoluta*, the dsRNA molecules were administered by sucrose droplet feeding [28,37]. A single second instar larva of *T. absoluta* (starved for 2 h) was carefully placed in a sterile 0.2 mL PCR tube, in which ten 0.25 μL droplets (1.5 mm in size is appropriate for larval feeding) had been placed (Figure 1B). According to our preliminary experiments this quantity of sucrose solution could support the development of the larva for a period of 4 d. The solutions used were: (a) sucrose only, (b) sucrose + ds*GFP* and (c) sucrose+ds*Ta-αCOP*. The final concentration of sucrose in the administered droplets was 0.5 M and that of dsRNA was 0.5 μg/μL. The PCR tube opening was covered with parafilm and pin holes were made on it to allow adequate ventilation. These PCR tubes were placed on a wet cotton bed in a 12-cm-diameter Petri dish and kept at 25 ± 1 °C, 65 ± 5% RH, and 16 L:8 D photoperiod. Larvae were allowed to feed for 4 d in the PCR tubes; upon completion of the 4-day-feeding period, with the help of a fine brush, they were carefully placed individually on a freshly-cut tomato leaflet supported by a wet sterile cotton in a Petri dish (Figure 1B).

The amount of time required for each larva to find a suitable feeding site on the tomato leaflet and insert its head in the mesophyll was recorded over time. Then, each larva was allowed to feed on the leaflet in the dish. The effect of the dsRNA application on larval survival was recorded daily until pupation. A larva was considered dead when it was not able to move back to a ventral position after being placed on its dorsum within one minute [38]. In addition, the developmental period and pupal weight for each larva were recorded. Each pupa was weighed using an analytical balance (Kern ACS 80-4; Balingen, Germany). Finally, adult survival was monitored for one week after emergence from the pupae. During this period the adults were kept in cages with tomato plants and were provided with sugar solution as food supplement. The experiment was repeated thrice. 14 larvae/treatment were used in each experiment.

### 2.4. Gene Expression Analysis 

Three independent biological replicates were employed for gene expression analysis. Four *N. tenuis* adult individuals per treatment were selected immediately after the completion of the 4-day-feeding period and RNA was extracted as described above. Reverse transcription (RT) was performed by employing oligo-dT and random primers. In brief, 2 μL from each RNA sample was mixed with 0.5 μL of oligo-dT (100 μM), 0.5 μL of random primers (100 μM) and 12 μL of RNase-free water. The PCR tubes were placed at 65 °C for 5 min and quickly transferred on ice. Then 5 μL of hot mix (2 µL 10× RT buffer with DTT, 1 µL dNTPs [10 mM], 0.5 µL FIREScript reverse transcriptase [Solis BioDyne, Tartu, Estonia], 0.25 μL RNase inhibitor and 1.25 µL of RNase-free water) were added in each PCR tube. RT was performed at 27 °C for 10 min followed by 60 min at 37 °C to produce cDNA, then the enzyme was inactivated at 85 °C for 5 min and the tubes were stored at −20 °C. Semi-quantitative PCR reactions were carried out using the KAPA Taq DNA polymerase or the KAPA high fidelity (HiFi) DNA polymerase (Kapa Biosystems, Cape Town, South Africa) following the manufacturer’s instructions. Quantitative PCR (qPCR) analysis was performed employing the 5X HOT FIREPol EvaGreen qPCR Supermix (Solis BioDyne), in a StepOnePlus Real-Time PCR System (Applied Biosystems, Forster City, CA, USA). Relative quantification of gene expression was carried out using the 2^−∆∆CT^ method, as described by Schmittgen and Livak [39].

### 2.5. Statistical Analysis of Data

Significant differences in the quantity of the endogenous *αCOP* gene expression level in *N. tenuis* nymphs fed with sucrose only, sucrose+ds*GFP*, sucrose+ds*Ta-αCOP*, or sucrose+ds*Nt-αCOP* were determined by Student’s *t*-test (*p* < 0.05) performed in a pairwise manner. For normalization purposes, *ATPB* was used as the internal control. The mean survival curves of *T. absoluta* larvae and *N. tenuis* individuals on the different treatments were compared by log-rank (Mantel-Cox) test (*p* < 0.0001) with GraphPad Prism version 8.2.0 software (San Diego, CA, USA). For the study of sublethal effects of dsRNAs, each *N. tenuis* nymph was considered as one replicate. The data of the three independent experiments were pooled and one-way ANOVA was performed to analyse the predation rate of *N. tenuis*. In a similar manner, the time required by each *T. absoluta* larvae to initiate tunnel mining was investigated. The mean values of all treatments were compared using the Tukey’s HSD test (α = 0.05) by statistical package JMP 14.1.0. 

## 3. Results

### 3.1. Effects of Oral Feeding of dsNt-αCOP in N. tenuis

**Figure 2:**
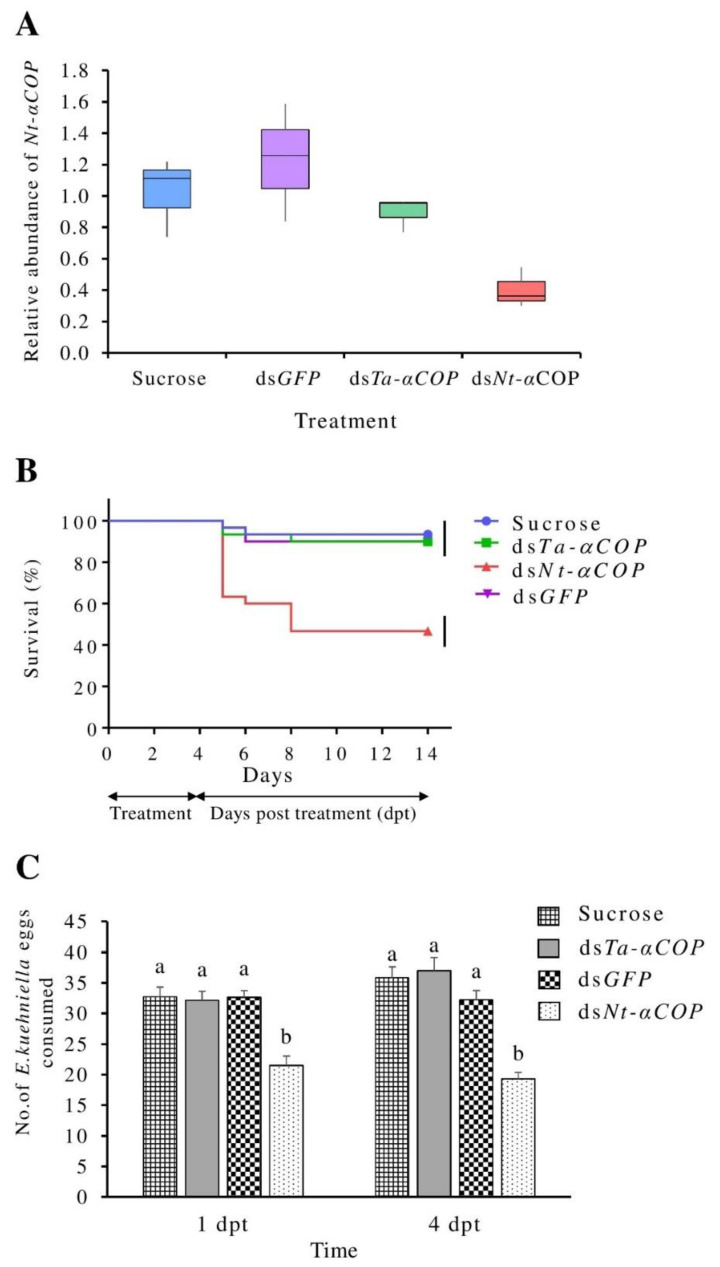
Effect of homologous and non-homologous dsRNA on survival and predation rate of *N. tenuis*. (**A**) Relative quantification of the endogenous *Nt-αCOP* gene expression by RT-qPCR. The treatments tested were sucrose, sucrose+ds*GFP*, sucrose+ds*Ta*-*αCOP*, and sucrose+ds*Nt-αCOP*. Results were obtained from three biological replicates. For normalization, *ATPB* was used as the internal control. Relative expression values were obtained using the 2^−∆∆CT^ method. For statistical analysis, the Student’s *t*-test was employed. The expression levels of *Nt-αCOP* in ds*Nt-αCOP* treatment significantly differ (*p* < 0.05) in comparison to the other three treatments. (**B**) Mean survival curves of *N. tenuis* fed from 0 to 4 d on sucrose, sucrose+ds*GFP*, sucrose+ds*Ta-αCOP*, sucrose+ds*Nt-αCOP* and subsequently fed with *E. kuehniella* eggs for 10 days on a tomato leaflet. Curves terminating at the different vertical bars are significantly different according to the log-rank test (*p* < 0.0001). (**C**) Number of *E. kuehniella* eggs consumed per *N. tenuis* individual at 1 and 4 dpt, after feeding for 4 d on sucrose, sucrose+ds*GFP*, sucrose+ds*Ta-αCOP*, sucrose+ds*Nt-αCOP*. Columns followed by different letter differ significantly (*p* < 0.05).

*αCOP,* an essential eukaryotic gene, was selected as the gene target in the present study. DsRNA (ds*Nt-αCOP*) targeting the *αCOP* gene (*Nt-αCOP*) in *N. tenuis* was produced (Figure S1) and fed to *N. tenuis* nymphs. ds*Nt-αCOP*-treated *N. tenuis* showed a mean reduction of *Nt-αCOP* transcript levels by 61% and 67% in comparison to the sucrose- and the ds*GFP*-treated controls, respectively (sucrose-treated: *p* = 0.010; ds*GFP*-treated: *p =* 0.011) (Figure 2A). This confirmed that the oral exposure of *N. tenuis* to gene-specific dsRNA can lead to RNAi-mediated gene knockdown. 

The successful gene-silencing of *αCOP*, via RNAi, has been reported to significantly reduce survival in other insects such as *Nezara viridula* (L.) (Hemiptera, Pentatomidae) [40], *Drosophila suzukii* (Matsumura) (Diptera, Drosophilidae) [36], and *Brassicogethes aeneus* (Fabricius) (Coleoptera, Nitidulidae) [41]. In our study, significantly higher mortality of 57% was recorded at 4 dpt (df = 2, F = 42.2, *p* < 0.0003) in ds*Nt-αCOP*-treated *N. tenuis* when compared to the ds*GFP* (10% mortality), and the sucrose (7% mortality) controls (Figure 2B).

Another important parameter that was examined at the organism level was the predation rate (proxy for sublethal effect) of *N. tenuis*. At 1 and 4 dpt, a significantly lower (df = 7, F = 11.12, *p* < 0.0001) predation rate (21.5 and 19.3 eggs consumed/adult, respectively) was recorded in *N. tenuis* adults fed with ds*Nt-αCOP* when compared to the sucrose- and ds*GFP*-treated controls (Figure 2C).

### 3.2. No Significant Cross-Silencing Effects of dsTa-αCOP on N. tenuis

Ds*Nt-αCOP*-treated *N. tenuis* showed a mean reduction of *Nt-αCOP* transcript levels by 55% in comparison to ds*Ta-αCOP*-treated *N. tenuis* (*p* = 0.004) (Figure 2A). In addition, no significant reduction in the transcript levels of *Nt-αCOP* was observed (*p* > 0.05) in ds*Ta*-*αCOP*-treated *N. tenuis* when compared to either of the controls (ds*GFP*- or sucrose only-treated *N. tenuis*) (Figure 2A). These observations correlated with the low level of sequence complementarity between the selected *Ta-αCOP* region (used for ds*Ta*-*αCOP* production) and its ortholog *Nt-αCOP* in *N. tenuis* (Figure S2), suggesting that ds*Ta*-*αCOP* does not trigger any significant cross-silencing effect against the ortholog *αCOP* gene in *N. tenuis*.

Moreover, no significant difference (*p* > 0.78) in the mortality was recorded at 10 dpt in *N. tenuis* that were treated with ds*Ta-αCOP* (mortality: 10%) when compared to the ds*GFP* (10% mortality) or sucrose (7% mortality) controls (Figure 2B). This result correlated with the observation that the transcript level of *Nt-αCOP* did not significantly change in these treatment groups. On the other hand, the 10% mortality in ds*Ta-αCOP*-treated *N. tenuis* is significantly lower (*p* = 0.0001) than the 57% mortality recorded in ds*Nt-αCOP*-treated *N. tenuis*. This indicates that the exogenous application of ds*Ta-αCOP* might not cause lethal effects in *N. tenuis*.

Lastly, ds*Ta-αCOP*-treated *N. tenuis* showed no significant difference in predation rate at 1 and 4 dpt when compared to any of the controls (ds*GFP-* and sucrose only treated groups) (Figure 2C). Most importantly, the predation rate of ds*Ta-αCOP*-treated *N. tenuis* was significantly higher (32.1 and 37 eggs consumed/adult on 1 dpt and 4 dpt, respectively) than in ds*Nt-αCOP*-treated *N. tenuis* (21.5 and 19.3 eggs consumed/adult on 1 dpt and 4 dpt, respectively). In sum, our data might signify that the performance of the predator *N. tenuis* was not negatively affected by the dsRNA targeting the prey *T. absoluta*.

### 3.3. Oral Delivery of dsTa-αCOP to T. absoluta Can Cause Lethal to Sublethal Effects 

**Figure 3:**
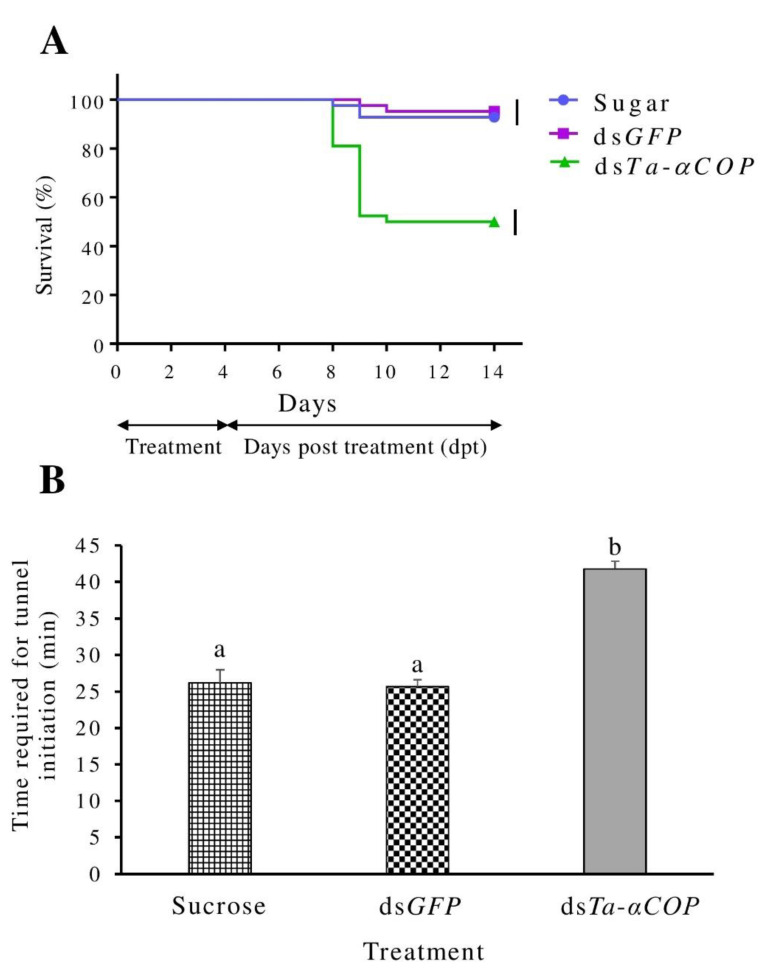
Effect of ds*Ta-αCOP* application on L2 larvae of *T. absoluta* through sucrose droplet oral delivery. (**A**) Mean survival curves of second-instar larvae of *T. absoluta* fed for 4 d on sucrose, sucrose+ds*GFP*, sucrose+ds*Ta-αCOP*, and subsequently fed for 10 days on a tomato leaflet. Curves terminating at the different vertical bars are significantly different according to the log-rank test (*p* < 0.0001). (**B**) Time required (mean ± SE) to initiate tunnel mining by second-instar larvae of *T. absoluta* fed for 4 d on sucrose, sucrose+ds*GFP*, and sucrose+ds*Ta-αCOP*. Columns followed by different letter differ significantly (*p* < 0.05).

A significantly higher mortality rate was observed in *T. absoluta* larvae that were fed with ds*Ta-αCOP* when compared to either the sucrose only- or the ds*GFP*-treated larvae. By 5 dpt, mortality had reached an average of 50% for ds*Ta-αCOP*-treated larvae when compared to the ds*GFP*-treated control (df = 2, F = 85.69, *p* < 0.0001) (Figure 3A). No significant difference in survival was recorded between the sucrose only- and the ds*GFP*-treated groups. In addition to survival, the amount of time required by *T. absoluta* larvae to penetrate the leaf tissue was also evaluated. Our results indicated that ds*Ta-αCOP*-treated larvae required a significantly (*p* < 0.0001) longer period of about 41.7 min to penetrate the leaf tissue when compared to the ds*GFP*-treated control that required 25.7 min (Figure 3B). This was about double the time required for tunnel initiation for the ds*Ta-αCOP*-treated larvae, indicating possible sublethal effects following oral exposure to gene-specific dsRNA. Nevertheless, 100% of the surviving larvae (sucrose: *n* = 39; ds*GFP*: *n* = 40; ds*Ta-αCOP*: *n* = 21) successfully pupated and emerged as adults in all treatments and further survived for 7 d after emergence.

## 4. Discussion

Our data indicated that dsRNA targeting *Ta*-*aCOP* of the pest, *T. absoluta*, did not cause any lethal nor sublethal effects in its biological control agent, *N. tenuis*. Furthermore, the predatory behaviour of *N. tenuis* was not affected at 1 and 4 dpt after four days of exposure to ds*Ta*-*aCOP*.

For the first time, it was shown that the application of dsRNA targeting the *Ta-αCOP* gene caused 50% mortality in *T. absoluta*. Camargo et al. [10] reported 50% and 43% mortality in *T. absoluta* through silencing of the genes *V-ATPase* and *AK*, respectively. Mortality percentages of 45%, 46%, 49% and 72% were observed when larvae were exposed to dsRNA designed to target the genes *CHI, JHP, COE* and *AK*, respectively [11]. Therefore, the gene *αCOP* can cause relatively significant mortality in *T. absoluta* within a week. This agrees with the lethal effects of dsRNA targeting the gene *aCOP* designed for other pest insects such as the sweet potato weevil, *Cylas formicarius* (F.) (Coleoptera, Brentidae) [42] and pollen beetle *Brassicogethes aeneus* F. (Coleoptera, Nitidulidae) [41]. In addition, its compatibility with *N. tenuis* indicates that *Ta-αCOP* is a potentially valuable gene to be employed in further developments of RNAi-based strategies against *T. absoluta.*

Along with the mortality in ds*Ta-αCOP*-fed larvae of *T. absoluta*, the time required to initiate feeding into the mesophyll of the tomato leaflet was nearly twice when compared to the control (ds*GFP*-treatment), which demonstrated an RNAi-mediated sub-lethal effect on the larvae. In IPM such delays are of particular importance because the treated larvae would be exposed for a much longer period either to predation risk by the foraging biological control agents or to residues of contact insecticides applied on the leaf. In the latter case, there may be a reduction of pesticide use due to the increased efficacy of the pesticide application. Therefore, the evaluation of non-lethal effects of RNAi on *T. absoluta* may also reveal synergies with the other IPM tactics and their significance in *T. absoluta* control should be investigated in future studies. The advantages of RNAi-mediated pest control indicate its potential for future field application [43,44,45]. However, a fundamental step in this direction is the development of appropriate risk assessment tools to generate reliable data for registration purposes [29,46,47,48].

Notably, in previous studies [49,50,51] RNAi was reported as ineffective or of low effectiveness in insects belonging to several orders, including hemipteran. Therefore, here, we targeted an endogenous gene in *N. tenuis* and demonstrated, for the first time, that RNAi is functional in *N. tenuis*. This is an important finding, showing that future efforts made in the direction of RNAi-based pest management should consider possible non-target effects on *N. tenuis*. This is particularly relevant because *N. tenuis* is a generalist predator since, apart from *T. absoluta*, it is also widely recruited against key hemipteran pests of tomato such as the whiteflies *Trialeurodes vaporariorum* (Westwood) or *Bemisia tabaci* (Gennadius) [52].

Our study confirms that the oral delivery method is practical with a chewing insect, namely *T. absoluta,* and a piercing insect such as *N. tenuis*. Therefore, this method can be used in laboratory risk assessment studies of RNAi on non-target insects [30,46]. Its effectiveness also reflects its potential ease in applicability considering that field application of tailor-made dsRNA pesticides most likely will be administered through spraying in future agricultural practices.

Taken together, our findings offer new insights in the potential of RNAi as an advanced method for sequence-based pest management strategies against *T. absoluta*. The application of ds*Ta-αCOP* causes mortality to *T. absoluta* and is safe for its biological control agent *N. tenuis*. Therefore, RNAi has the potential to be compatible with the utilization of *N. tenuis* in an efficient IPM strategy against *T. absoluta*; however, more field-realistic studies should elaborate this further.

## Figures and Tables

**Table 1:** Primers designed in this study for in vitro production of dsRNA and gene expression analysis in *Nesidiocoris tenuis* and *Tuta absoluta*.

Name	Type	Sequence (5′–3′)	Product Size (nt)	Target Species	Purpose
ds*Nt-αCOP* (joined to T7 promoter)	F R	TAATACGACTCACTATAGGG CACACTGCCCCTGATCGTAT TAATACGACTCACTATAGGG GTCGAGTTTACGCAGGAAGC	391	*N. tenuis*	dsRNA production
qPCR*-Nt-αCOP*	F R	GGGAGGACTCGAAGAACATTT GATCGTGCCCTTCCAAGAC	95	*N. tenuis*	qPCR
qPCR*-Nt-ATPB*	F R	CATACGCCAAGGGAGGTAAA CTGGGTGAAACGGAAAATGT	356	*N. tenuis*	qPCR
ds*Ta-αCOP* (joined to T7 promoter)	F R	TAATACGACTCACTATAGGG CCGTTTTCATCACAGGTCT TAATACGACTCACTATAGGG CGGTCATGGCCACTAAGAAT	505	*T. absoluta*	dsRNA production

## Supplementary Materials

The following are available online at https://www.mdpi.com/2075-4450/12/4/274/s1, Figure S1: In vitro synthesis of dsRNA for application in *T. absoluta* and *N. tenuis*, Figure S2: DNA sequence alignment between the region of *αCOP* from *T. absoluta* used for dsRNA production and the respective region of *αCOP* from *N. tenuis*.
