# Supplementary material for: dsRNA-Mediated Pest Management of Tuta absoluta Is Compatible with Its Biological Control Agent Nesidiocoris tenuis
Source: Insects. 2021 Mar 24;12(4):274. doi: 10.3390/insects12040274 (PMC8063791; doi:10.3390/insects12040274)
Supplement: Supplementary file 1 [file insects-12-00274-s001.pdf]

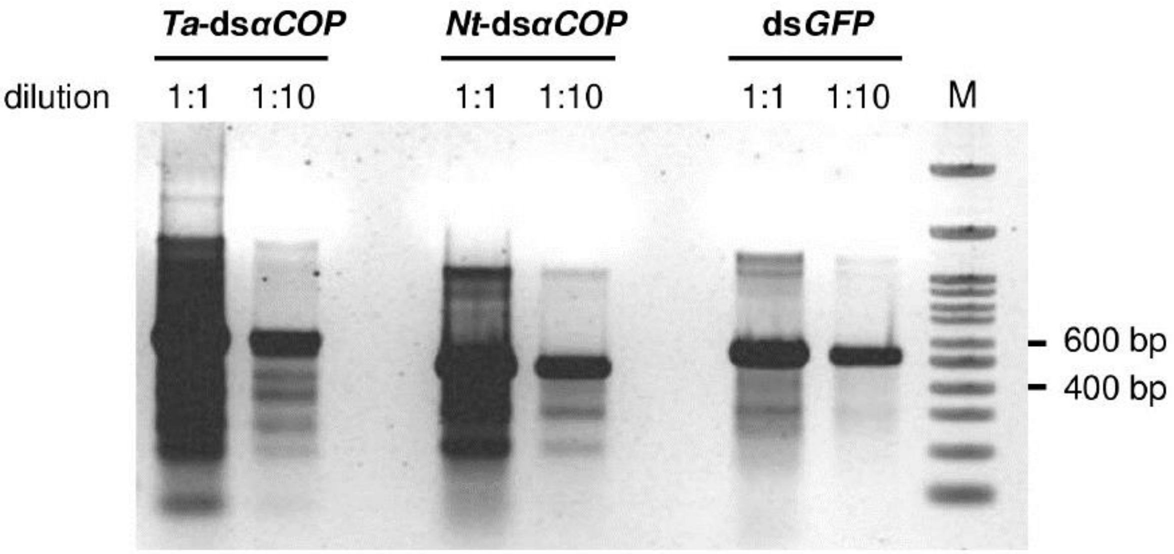

Figure S1. In vitro synthesis of dsRNA for application in *T. absoluta* and *N. tenuis*.

```
αCOP_N.tenuis     CCttggaTTTTggTgA---GctTgCATAgcGGggttATTcAActtTGGGAcTACCggatg
αCOP_T.absoluta   CC----gTTTTcaTcAcagGtcTtCATAatGGttcaATTaAAgccTGGGAtTACCaatca

αCOP_N.tenuis     tgcaCaCTgtTGgAcaAgTTcgATGAgCAcGAcGGccCgGTaAGAGgCATctgTTTCCAT
αCOP_T.absoluta   aatgCtCTtaTGcAtgAaTTtaATGAcCAtGAtGGttCaGTcAGAGcCATtacTTTCCAT

αCOP_N.tenuis     aCcCAgcAGccGcTTTTcgTtTcAGg--AGGAGAcGATtAcAaAATcaaAgTaTGGaAcT
αCOP_T.absoluta   cCtCA--AGgtGaTTTTttTaTaAGtgcAGGAGAtGATaAaAtAATacgAcTtTGGgAtT

αCOP_N.tenuis     AcAaAcatcGAcGAtgcaTCTttActttGTT---gGGtCActtgGAcTacATcCGTaCga
αCOP_T.absoluta   AtAcA---aGAaGAactcTCTcaAaaaaGTTtaaaGGaCAtacaGAtTttATtCGTgCtc

αCOP_N.tenuis     ccatgTTcCAtCaGgagtAcCCgTGGaTccTcAGcgCcTCCGAcGATCAgACCATTcGtA
αCOP_T.absoluta   ttgacTTtCAcCcGactaAgCCcTGGtTtgTtAGttCtTCCGAtGATCAaACCATTaGaA

αCOP_N.tenuis     TTTGGAAT--------TGGCAAAgccgtactTGcaTttGtgtgctCACcGGgCAcaatCA
αCOP_T.absoluta   TTTGGAATtttatgacTGGCAAA--------TGttTagGaacagcCACtGGtCAttcgCA

αCOP_N.tenuis     TTACgTcATGtgcGctcaATTccacccTacAgATGAcAtcgtCgTttcA---GcATCgTT
αCOP_T.absoluta   TTACaTaATGgcaGtaagATT------TttAaATGAaAattcCtTaatAagtGgATCtTT

αCOP_N.tenuis     gGAcatgaCcgTccGAGTcTGGgatatatcggggctgaggaaaaagaacGTTGctccggg
αCOP_T.absoluta   aGAtcaatCttTaaGAGTtTGGa--------------------------GTTGt------

αCOP_N.tenuis     cccgGgAGGaCTcgaaGAacATttgAAGAAcccatcggccacGgaCctTTTcGgTCaggC
αCOP_T.absoluta   ----GaAGGtCTtattGAtaATacaAAGAA------------GagCacTTTtGtTC---C

αCOP_N.tenuis     TgacgcTGTggTcAAACAcgTctTggaaGGgCAcGAtCGt
αCOP_T.absoluta   TagtatTGTtaTaAAACAaaTtcTtagtGGcCAtGAcCG-
```

Figure S2. DNA sequence alignment between the region of *αCOP* from *T. absoluta* used for dsRNA production and the respective region of *αCOP* from *N. tenuis*.
